# Implementing mass dog rabies vaccination through a community-based continuous approach: A socio-anthropological process evaluation

**DOI:** 10.1371/journal.pntd.0014091

**Published:** 2026-06-30

**Authors:** Christian Tetteh Duamor, Felix Lankester, Katie Hampson, Ahmed Lugelo, Kennedy Selestin Lushasi, Maganga Sambo, Joel Jackson Changalucha, Anna Czupryna, Sally Wyke

**Affiliations:** 1 Environmental Health and Ecological Sciences Thematic Group, Ifakara Health Institute, Dar es Salaam, Tanzania; 2 School of Biodiversity, One Health and Veterinary and Life Sciences, University of Glasgow, Glasgow, United Kingdom; 3 Global Animal Health Tanzania, Arusha, Tanzania; 4 Paul G. Allen School for Global Health, Washington State University, Pullman, Washington, United States of America; 5 Department of Veterinary Medicine and Public Health, college of Veterinary Medicine and Biomedical Sciences, Sokoine University of Agriculture, Morogoro, Tanzania; 6 School of Social and Political Sciences, University of Glasgow, Glasgow, United Kingdom; Colorado State University, UNITED STATES OF AMERICA

## Abstract

**Introduction:**

Mass dog vaccination to control rabies usually involves centralized planning and team-led annual campaigns. We evaluated the implementation processes of a decentralized, community-based continuous approach to mass dog vaccination (CBC-MDV), delivered as an arm of a randomized controlled trial (RCT) across the Mara region, Tanzania. We employed the socio-anthropological framework to assess the implementation process of the CBC-MDV approach.

**Methods:**

We embedded a mixed method process evaluation into the RCT and conducted in-depth interviews with district and village-level implementers, focus group discussions with ward-level implementers and community leaders and members, non-participant observation of the campaigns, and an audit of the vaccination campaign activities. We analyzed the resulting data following the five domains of the socio-anthropological framework.

**Findings:**

Refining the five domains, we report that 1) **Environmental context** affected CBC-MDV campaigns, which were interrupted by rainy and farming seasons, school schedules, market days and funerals, whilst village size and routes of access reduced their reach. 2) **Social difference and pressures, and community involvement** influenced delivery; cultural celebrations and social statuses of implementors hindered delivery, while by-laws and perceived rabies risk improved delivery. Community involvement facilitated implementation, but there was a need to strategically select who did what and how. 3) **Strategies and incentives** affected implementers, who were motivated by training and resources provided, and delivered 88% of components as planned. 4) **Socio-materiality and resilience of intervention strategies and tools** made it feasible to deploy the required equipment across a wide geographic scale, with adequate vaccination opportunities, but implementers found house-to-house and on-demand strategies demanding and less effective than central point delivery. 5) **Governance** also affected delivery with closer supervision by veterinary officials and community leaders, and co-reflexive appraisal of implementation with communities constrained by resource limitations.

**Conclusions:**

CBC-MDV with local storage of dog vaccine can be deployed across the rural landscape of Tanzania. Deeper involvement of communities in planning when and how campaigns are conducted, and in the appraisal of outcomes can significantly address barriers to dog-owner participation. The socio-anthropological framework provides a useful framing for understanding intervention delivery but not refinement for use cases.

## Author summary

Mass dog vaccination campaigns in most rabies-endemic countries are planned and delivered by veterinarians annually without involving local communities. An alternative approach that involves communities, storing vaccines locally, and enabling several rounds of campaigns to be carried out within a year, was tested in the Mara region of Tanzania. We used a socio-anthropological framework to explore how the implementation of the strategic components of this community-based approach was affected by environmental and socioeconomic factors; what motivated implementers; the usefulness and resilience of the intervention strategies and tools; and how implementation was coordinated. We collected data through interviews of implementers and community members, observation of campaigns and review of implementation records. We found that rainy and farming seasons, school and market days, cultural celebrations and funerals affected campaign schedules; whilst difficult terrain, by-laws, perceived rabies risk, and community involvement impacted reach of the campaigns. With time, the implementers delivered most components as planned; it was feasible to deploy the tools and strategies in the context; supervision of campaigns by community leaders and veterinary officials was limited. We conclude that the active involvement of communities in planning when and how vaccination campaigns are conducted and evaluated can significantly improve dog-owner participation.

## Introduction

Rabies is a deadly zoonotic virus that affects mammals and more than 95% of human cases are contracted from domestic dogs [1,2]. The disease is entirely vaccine-preventable in humans and animals, with evidence that sustaining dog vaccination coverage interrupts transmission in dog populations, and prevents spillover to wildlife and humans [3–5]. However, across most of Africa and Asia, the regions of the world that share the vast majority of the human mortality burden [6], mass dog vaccination campaigns have not been conducted at scale [7–9]. Rather, mass dog vaccination tends to be undertaken as localized projects by research or charitable missions. Given this, and the lack of funding available for mass dog vaccination, there is a need to investigate approaches for scaling up dog vaccination at low cost.

The effectiveness of any scaled-up intervention depends upon its implementation, which can be negatively impacted when the details such as the ‘what’, ‘how’, ‘when`’ and ‘by whom’ are largely defined by technocratic perspectives, without the participation of the targeted communities [10,11]. This results in implementation strategies that poorly fit the environmental and sociopolitical realities of targeted communities [10,11], overstretch available resources [12–14], are not implemented correctly, and fail to reach their full potential [7]. For example, where dog vaccination has been implemented in Tanzania, coverage is often patchy [8] due to inadequate dog owner participation [8,15,16] and limited community support for the process [17].

Challenges affecting programming and implementation can often be traced to the priorities of implementing organizations and funding [10] with improvements in effectiveness potentially arising when adequate funds and time are allocated to tailoring the intervention strategies to local contexts. Therefore, both implementing and funding agencies must consider the time and budgetary space needed to translate proofs of concept into local contexts and for communities to own the resulting intervention strategies. These considerations, in addition to the determination that Nobivac dog rabies vaccines are thermotolerant, provided context for the development of a community-based continuous mass dog vaccination (CBC-MDV) approach [18–20] in Tanzania.

CBC-MDV was designed to provide communities with continuous access to dog vaccination, and therefore to ensure coverage above the critical threshold for herd immunity is sustained all year around. A target that is difficult to achieve with centralized approaches that are pulsed, tending to occur annually [20]. CBC-MDV was initially piloted in nine wards across three districts in Tanzania with varied environmental, cultural and socioeconomic settings [17,20]. Following an optimization process, the approach was scaled up to 56 wards across all six districts of the Mara region as part of a cluster Randomized Controlled Trial (RCT).

We conducted a mixed method, exploratory process evaluation focusing on the CBC-MDV arm of the RCT. We analyzed the resulting data adapting the socio-anthropological framework proposed by Bardosh [10] to assess how: i) the environmental context; ii) social difference and pressures, and community involvement; iii) strategies and incentives of implementers; iv) utility and resilience of intervention tools under field conditions and v) governance of CBC-MDV, impacted the implementation process. Finally, we explored the usefulness of the socio-anthropological framework in describing implementation success of CBC-MDV.

## Methods

### Ethics statement

The protocol for this study was reviewed and approved by the Institutional Animal Care and Use Committee, Washington State University [Approval No. 04577 – 001], the Tanzania National Medical Research Institute [NIMR/HQ/R.8a/Vol.IX/2788], the Tanzania Regional Administration and Local Government [AH.213/420/01] and the Ifakara Health Institute [IHI/IRB/No:024-2018]. Administrative permissions were sought from the Mara regional and Buda, Serengeti, Musoma, Rorya, Butiama and Tarime district veterinary offices and the leaderships of the wards and villages involved in the study. Participants received information aim and procedures of the study, they were then allowed time to ask questions and agreed to participate by signing a formal written consent form.

### Study setting

The RCT was conducted in 112 wards across the six districts of the Mara region of Tanzania, between Lake Victoria and Kenya. These districts are home to ethnic groups primarily engaged in pastoral, agro-pastoral, fishing and trading activities. Dog ownership is common, with livestock-owning households tending to own more dogs [21–23].

**Fig 1:**
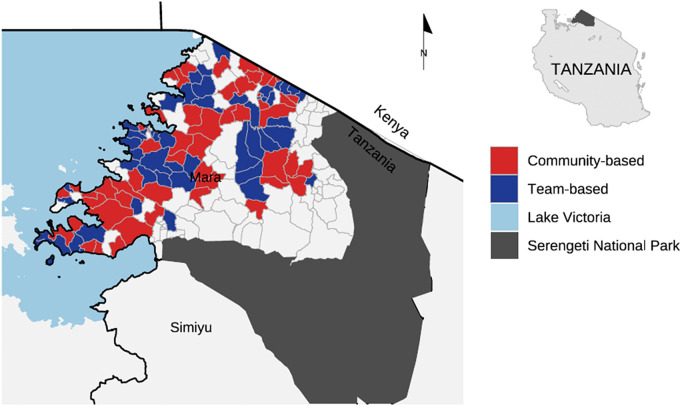
Map of Mara Region of Tanzania. The shapefiles are freely available at: https://www.nbs.go.tz/uploads/statistics/documents/en-1714652282-TANZANIA_2022PHC_WARD_SHAPEFILES.zip.

In Tanzania, administrative wards are composed of clusters of three to four villages, with each village divided into sub-villages. The RCT excluded wards adjacent to the Serengeti National Park where dog vaccination had since 2012 been carried out through annual campaigns conducted through a government-research project partnership (Fig 1). This process evaluation focused only on delivery of strategy components of the CBC-MDV arm of the trial, involving 56 wards.

### Description of team-led and CBC-MDV approaches to mass dog vaccination

The centralized, team-led approach has been the status quo for delivering mass dog vaccination campaigns in Tanzania and involves vaccination teams from district livestock offices organizing campaigns in each community once a year, described extensively by Lugelo and co [20]. The decentralized, CBC-MDV approach was made possible by the discovery that the Nobivac dog rabies vaccine remained potent following extended non cold chain storage and designed to provide continuous access to dog vaccination in rural communities in Tanzania by storing vaccines at ward level and conducting quarterly central point campaigns as well as providing vaccination upon request from dog owners. CBC-MDV was designed through iterative multi-sector stakeholder workshops and piloted for a year in nine wards from three districts of the Mara region [17]. S1 File presents a full description of CBC-MDV following the TIDieR template [24].

The implementation of CBC-MDV was coordinated at the district level by District Livestock Field Officers (DLFOs) who coordinated the supply of vaccines and equipment to the vaccination teams and supervised the campaigns. CBC-MDV was implemented at the ward level by livestock field officers (termed Rabies Coordinators (RCs)), who organized and coordinated the village-level campaigns in their respective wards and were responsible for inoculating dogs. At the village-level, CBC-MDV was supported by Village Chairpersons, partisan representatives elected by a simple majority (termed One Health Champions – OHCs) who assisted with the organization of campaign activities in their respective villages.

Implementers from each district were trained in a three-day workshop which focused on the concept of CBC-MDV and skills for its delivery, and practical sessions that were carried out in a selected village of each district.

### Evaluation design

We conducted a mixed method, cross-sectional exploratory process evaluation of the CBC-MDV arm of the RCT. The RCT took place between July 2020 to June 2023 and the process evaluation between March 2023 and May 2023. In the RCT, 56 wards were randomly assigned each to the team-led and CBC-MDV arms. Another study compared the vaccination coverage and operational costs [25] for these two arms.

This study focused on assessing what impacted the implementation processes of the CBC-MDV approach, guided by adapted constructs of the socio-anthropological framework [26] (Table 1). The framework employs five thematic domains to explore the determinants of effectiveness of interventions aiming to control Neglected Tropical Diseases. Other studies that have applied this framework suggest it provides a useful structure for describing the context of implementing One Health interventions at the community level [10,27]. We also assessed the usefulness of the framework.

**Table 1:** Constructs of the socio-anthropological framework [10] and how they were operationalized in this study.

Domain (adapted *name*)	Original construct explanation	Adaptation of the construct in our study
1. Terrain of intervention (*Environmental context of implementation*)	How seasonal, environmental and economic patterns and geographic characteristics of the environment in which the intervention was delivered affected the delivery process and effectiveness of the intervention	Any natural or socioeconomic characteristics of the environment that facilitated or hindered the delivery process (e.g., season, accessibility of settlements, socioeconomic events, livelihood activities etc.)
2. Social difference and community agency (S*ocial difference and pressures, and community involvement*)	How differences in wealth, ethnicity, livelihoods, power, knowledge, cultural norms and needs, capacities and constraints influence interest in and adoption of the intervention, and simplified local narratives about why people should comply with the interventions	How differences in social status and relationship with dogs influenced people’s participation in the vaccination campaigns. What social pressures such as by-laws and perception of risk of rabies influenced participation in the campaigns How community involvement in general and the designation of village elected chairpersons as One Health Champions (local coordinators of the campaigns) affected the implementation process
3. Strategies and incentives of field staff (S*trategies and incentives of implementers*)	How field staff delivered the intervention and what motivated them (skills, motivations, capabilities and support)	How and why the implementation teams delivered CBC-MDV the way they did (e.g., whether they delivered it as intended and reasons for variation). Their motivations for performing or not performing certain activities (e.g., training, financial, support and feedback systems)
4. The socio-materiality of the technology/intervention (*Socio-materiality and resilience of intervention strategies and tools*)	The characteristics of intervention tools and how this mediated adoption, delivery and use patterns	The characteristics of CBC-MDV strategies and the tools deployed in its delivery (continuous vaccination, local storage of vaccine, passive cooling clay pots and their temperature loggers) and how those supported the implementation process
5. Governance (G*overnance of CBC-MDV delivery*)	The influence of policy structures, bureaucracy, politics and the utilization of knowledge on the success of implementation	How CBC-MDV activities were supervised and coordinated by district veterinary authorities and the ideals of CBC-MDV which could be mainstreamed and sustained

### Data collection

#### In-depth interviews and focus group discussions.

To investigate the experiences of implementers and community members concerning the delivery of CBC-MDV, we conducted in-depth interviews (IDIs) with the DLFOs and OHCs. We conducted focus group discussions (FGDs) involving RCs and community members. While all DLFOs were selected for the IDIs, OHCs and participants in the FGDs were purposively selected to ensure representation of leadership, age groups and genders across the wards that delivered CBC-MDV.

The IDIs and FGDs were conducted between March and June of 2023, year three of the three-year RCT. Once participants had consented to participate, the interviews were conducted in person in Swahili by an experienced interviewer. The IDIs and FGDs lasted approximately 60 minutes and were recorded with an Olympus VN-541PC voice recorder. The topic guides covered involvement of local leadership in the implementation, how CBC-MDV components were delivered, how the implementation of CBC-MDV went and whether it was being established for delivery in the long term, community participation in the vaccination campaigns and mobilization of local resources to support CBC-MDV. The guides were revised after the first three interviews to ensure they were valid and administered efficiently.

#### Non-participant observations.

We conducted non-participant observations (NPO) (n = 40 hours) on five vaccination activities using a structured proforma guide to document what facilitated or limited the vaccination campaigns. The proforma guide recorded: i) what influenced the timing of, and turn out at, vaccination campaigns, such as the weather or social events on the day; (ii) how implementers delivered the CBC-MDV components and iii) the enhancers and barriers that they faced in organizing the vaccination campaigns.

#### Auditing CBC-MDV delivery.

Semi-structured questionnaires were administered to 45 out of the 56 RCs to explore their experiences in the first quarter of year three. We also examined their vaccination records to document how CBC-MDV was delivered and to document observed variations to the implementation manual and the reasons for these variations.

### Data analysis

Recordings from IDIs and FGDs were transcribed verbatim and translated into English. The transcripts and completed proformas from NPOs (field notes) were assigned unique identifiers and imported into NVivo 12 Plus version 20.7.1.1534. These data were coded by the first author (CTD), guided by a coding framework developed by CTD and the last author (SW). The initial coding framework was developed through familiarization with the data and basing on the priori concepts grounded in the socio-anthropological domains. CTD and SW independently applied the coding framework to five transcripts. They then met three times to clarify their understanding of applying the socio-anthropological framework and how each domain could be interpreted using our data (see Table 1 above). Once consensus was reached, the coding framework was applied to all transcripts by CTD.

The coded data extracts under each domain of the socio-anthropological framework from whichever data source were synthesized and the evidence summarized. The summaries within the domains described the experiences of implementers and communities with CBC-MDV delivery, how the CBC-MDV components were delivered and interacted with contextual elements, what facilitated or hindered the delivery, and how implementers and communities think CBC-MDV could be effectively and sustainably delivered beyond the funded research.

## Results

In-depth interviews were conducted with all six DLFOs and six out of 234 OHCs, one from each district where CBC-MDV was delivered, 12 FGDs were held, six involved 53 out of the 56 RCs and six involved 55 community members and leaders from 20 out of 234 villages involved in CBC-MDV. The implementation processes of 45 out of 56 RCs/ teams were audited and five non-participant observations were conducted on vaccination campaigns (Table 2).

**Table 2:** Respondents and numbers of in-depth interviews, focus group discussions, audits and observations conducted.

Districts	In-depth interviews		Focus group discussions		Audits	Observations
	DLFOs (N = 6)	OHCs (N = 234)	RCs/ Wards (N = 56)	Community Members & Leaders (N = 234)	RCs (N = 56)	Vaccination Teams (N = 56)
Bunda DC	1	1	1	1	5	2
Butiama	1	1	1	1	7	–
Musoma DC	1	1	1	1	9	–
Rorya	1	1	1	1	6	1
Serengeti	1	1	1	1	8	–
Tarime	1	1	1	1	10	2
Totals	6	6	6	6	45	5

N = Number of personnel or teams involved

### Domain 1: Environmental context of intervention

#### Seasonality.

CBC-MDV was impacted by seasonality: during the rains, when roads were less passable and some villages or sub-villages temporarily cut off, vaccinators sometimes had difficulty traveling to advertise and conduct campaigns.

*“[…] especially during the rainy season when some areas are inaccessible. We have to wait until June or July when the rain stops to vaccinate in those areas” (P9, RCs FGD, District 3).*

The non-participant observation data and reports of vaccinators corroborated that rain did sometimes interrupt the vaccination campaigns, causing the affected teams to reach fewer dogs on those days.

*“You go to vaccinate around 10 am and the rain starts. So, the dog owners wouldn’t come, and we had to reschedule” (P4, RCs FGD, District 4).*

Non-participant observations and interviews demonstrated that farmers were often busy during the rainy season, and either brought their dogs for vaccination after working on the farms or missed the campaign altogether.

*“Their priorities are set by the seasons because their survival often depends on food. During the rainy season, they need to farm, and no matter what, they will start with the farm before anything else. So, seasonality drives their actions” (DLFO IDI, District 4).*

#### Timing of social events.

Recurrent events such as school schedules, market days and funerals impacted campaigns. During the months when primary and secondary schools were in session, vaccinators tried to schedule most campaigns on weekends to enable children, who normally bring the dogs, to participate.

*“I face a lot of challenges at the village level because it is students who mostly bring dogs, so if your schedule falls on school days, you won’t get many dogs. It forces me to schedule on weekends or holidays, we perform better than on regular days” (P4, RCs FGD, District 6).*

Additionally, implementers reported that school sessions were effective platforms to advertise the campaigns.

*“When we announce in schools, the information spreads to a larger area than just walking around. Dogs are often friends of children in our communities, children know dogs better than parents” (P4, RCs FGD, District 4).*

The vaccinators had to ensure vaccination schedules did not conflict with regular socio-economic activities to promote participation.

*“For instance, our schedule is tailored to our surroundings because our economy largely relies on markets and traditional trading. So, you can’t set a schedule during those market days and expect it to work; you’d find yourself alone at the vaccination station” (P1, RCs FGD, District 5).*

In the context of Tanzania, because those who have died are normally buried within days, and because communities are highly connected, participation in vaccination campaigns was affected by funerals to which many people from across communities typically choose to attend.

*“In my ward, people are related, you might find that a person who died in one village has relations in other villages. So, if you schedule a vaccination and there’s a funeral, you might not have people” (P 4, RCs FGD, District 4).*

#### Geography.

Villages in the study area were typical of northern Tanzania in that they covered a large area with households spread far and wide. These factors posed challenges to OHCs during campaign advertising and documenting dogs that were missed in previous campaigns, increasing the transport needs and efforts required to conduct these activities effectively.

*“However, the main issue is how I can find the time and means to travel and identify the dogs in these areas. […], my village is geographically large” (OHC IDI, District 6).*

Owners expressed concerns about having to take their dogs long distances to reach the CBC-MDV vaccination points:

*“Considering the geography of our areas, even within one village with five sub-villages, it would be better to have a vaccination center in each sub-village […]; considering the distance, someone may bring only one or two dogs even though they have more due to the inconvenience caused by the dogs” (P9, Community Leaders & Members FGD, District 1).*

Some vaccinators also faced geophysical barriers in reaching certain communities; having to pass through mountainous terrains on foot or cross rivers, which took effort and time

*“The challenges we faced in some areas were related to transportation. Some places were inaccessible, so we had to leave vehicles behind and walk even to remote mountainous areas” (P1, RCs FGD, District 5).*

### Domain 2: Social difference and pressure, and community involvement

#### Social difference.

Participation was affected in communities having prolonged cultural celebrations such as puberty rites (circumcision).

*“The campaign faced challenges due to traditional issues, like circumcision. The neighborhood chairman might not be available because there could be ongoing circumcision ceremonies. People don’t prioritize vaccinations during festive occasions. So, when I identified such events, I tried my best to schedule around them” (P6, RCs FGD, District 1).*

Amongst household members, children tended to have stronger bonds with dogs. This had implications for who was best able to bring and restrain the dogs at vaccination points.

*“Yes, these dogs are used to children. […], taking them from one place to another, like three kilometers away to get vaccinated. So, the father might try to bring the dog but if it hears another dog barking, it changes its mind because it doesn’t know the father” (P1, RCs FGD, District 3).*

Differences in social statuses of RCs and OHCs created difficult working relationship between them: the OHCs wielded more social power and mostly older than the RCs, this made it difficult for the RCs to firmly instruct and direct the OHCs on how to perform their roles.

*“If you look at the project setup, it means this OHC will be under the [Rabies] Coordinator at the ward level. So, if you look at this chain of command, it does not auger well, that is, the personnel (civil servant) commanding a person who is a politician, do you see the challenge that arises?” (DLFO IDI, District 1).*

Other challenges associated with Village Chairpersons (OHCs) included their advanced age and physical frailty, which meant that they were sometimes not able to perform the duties effectively. Additionally, they were sometimes not available for vaccination activities because of political party and community responsibilities.

*“He didn’t even record things properly; he just issued cards. Sometimes he said the work was a waste of time. So, I worked with him for a while, but eventually, I had to replace him with someone from the sub-village. The work went smoothly after that” (P11, RCs FGD, District 2).*

Because of these issues, some RCs replaced the OHCs for lack of cooperation and instead worked with other persons including sub-village chairs.

*“Honestly, he has never reported anything related to vaccinations. So, I would go there myself, make calls, and visit the sub-villages. He was very relaxed, and at end of the month, he would inquire if they hadn’t paid yet. I replaced him with a more ordinary person” (P6, RCs FGD, District 6).*

The RCs also thought Sub-Village Chairpersons could better manage the campaigns workload.

*“However, if only one village chairman is responsible for all sub-villages, it will be very difficult because they have other responsibilities and no means of transportation. So, my suggestion is that the Sub-Village Chairpersons should be directly involved” (P1, RCs FGD, District 2).*

Some RCs believed Village Executive Officers (who are government employees) could perform better than Village Chairpersons as OHCs because of their executive powers to direct projects:

*“Another thing is the village executives. Those people are very important because they have the powers to apprehend those who did not vaccinate their dogs” (P1, RCs FGD, District 3).*

Additionally, some RCs believed that because Village Executive Officers do not have factions within the community and they could more easily mobilize people for the campaigns.

*“The key is to deal with the village executives, the role given to the Village Chairpersons should be transferred to the village executive. This is because, when the village executive makes a statement or declaration, a significant number of people respond. Because the village executive doesn’t have groups [factions], but the village chairperson has groups, so, when they speak, people respond differently” (P4, RCs FGD, District 6).*

On the other hand, in some cases, the political influence of OHCs was viewed as helpful in mobilizing the community to participate in campaigns.

*“Yeah, so when it comes to responsibility and involvement, we used to work well with them. They can motivate the local leaders to do this and that” (P3, RCs FGD, District 5).*

Conversely, working with OHCs was seen as an impediment to enforcing dog vaccination as the village leaders would not want to hurt their electoral fortunes by forcing people to vaccinate their dogs.

*“These are people who the community members elect and most of the time in some ways they are there to protect their community members, and control of diseases is an issue of law and not voluntary” (DLFO IDI, District 5).*

#### Social pressure.

Participation in the vaccination campaigns appeared to be influenced by the perceived risk of rabies within communities.

*“After the initial vaccination campaign, once the occurrence of rabies is reduced, people tend to become complacent. They no longer hear about rabid dogs, so some of them tend to neglect it” (OHC IDI, District 6).*

The presence of by-laws obliging dog owners to pay the full treatment costs when their unvaccinated dog bites someone was said to drive people to participate in the campaigns.

*“Though we haven’t implemented the legal actions we warned about, the threat alone of taking people to court and imposing fines has made people bring their dogs for vaccination” (P2, RCs FGD, District 5).*

#### Community involvement.

The implementers (DLFOs, RCs and OHCs) appreciated the importance of engaging and working with the community and local government leaders.

*“I introduced and explained the project, which helped overcome any resistance within the local government. The local government officials were able to communicate the project goals effectively down to the village level, as they had the mandate to do so. In the first year, we made good progress, exceeding our targets for the first phase. Involving local leaders and those who represent the community was crucial. They understand the local context better than us experts and can defend the project within the community” (P5, RCs FGD, District 2).*

Both RCs and community members believed the most important contributions that community members provided to MDV campaigns were to advertise, educate and mobilize their members to participate and enforce dog vaccination.

*“The main support the community leaders gave was mobilizing and educating the community on the reasons why they need to vaccinate their dogs” (DLFO IDI, District 6).*

### Domain 3: The strategies and incentives of implementers

#### Strategies of implementers.

The non-participant observation and audits of the CBC-MDV components showed that implementers delivered 88% (37 out of 42) as planned or slightly modified, compared to 69% during the pilot [28]. The CBC-MDV components were seen to be largely feasible to implement in the local context. However, there were some challenges with OHCs visiting houses after each round to document dogs that missed vaccination, and identify newly acquired dogs and puppies, and plan with RCs on how to vaccinate. These challenges arose primarily due to the need for transport (which was frequently not available or was costly) and OHCs not knowing where these dogs lived.

#### Incentives and disincentives of implementers.

The training given to OHCs and RCs was said to empower them with the skillset needed to deliver CBC-MDV components, operate the equipment and conduct dog vaccinations. This, they reported, made them confident and enthusiastic about their roles in the campaign.

*“I would say that the training, it made me fulfill my duties. Because when you receive training, you are quick to inform and educate someone when asked any questions” (OHC IDI, District 5).*

The RCs reported that the financial and material resources provided by the project greatly helped to facilitate the organizing and conducting of the campaigns.

*“Another important aspect I would like to highlight is resources. The fact that we had the vaccines and we could move around on motorcycles to reach remote areas has been very effective. Especially, for RCs and OHCs, this approach has helped tremendously” (P3, RCs FGD, District 4).*

RCs also reported that some OHCs became noncooperative due to dissatisfaction with their stipends. Some RCs also added that other community persons that they engaged also demanded compensation for their time, and for other costs incurred including meals and transport. During one implementation audit, an RC remarked:

*“Village chairpersons become noncooperative after few campaigns; (as a result) I normally work alone; they see the money as little. This is a mining area and a day’s wage can be 300,000 – 500,000TZS (about 120 – 200USD); the guy who used to do the transect said he can’t take 10,000TZS for it. A Sub-village chair requested 20,000TZS after helping to advertise the campaign a few days ago” (RCs Implementation audit, District 1).*

Implementers (DLFOs, RCs and OHCs) also pointed out that funds allocated for transport were insufficient. This affected the reach of their activities.

*“You find that the village has about five or six sub-villages, so you have to invite and follow them there. The costs of transportation are not completely covered in the budget. It means you have to cover the transportation costs yourself. So, the major issue is that certain costs need to be re-evaluated in implementing these activities” (P3, RCs FGD, District 5).*

Delays in replacement of equipment, supply of vaccination materials, and resolution of technical, financial and administrative issues frustrated implementers and dampened their morale.

*“The district officer tells you that the supplies are not there, they placed an order more than two weeks ago but the supplies haven’t arrived yet. So, there’s a delay in vaccine delivery” (P4, RCs FGD, District 6).*

### Domain 4: Socio-materiality and resilience of intervention strategies and tools

#### Impact of CBC-MDV strategies.

In month one of each year, central point campaigns were conducted at the village level. Subsequently, village or sub-village-level central point campaigns, house-to-house campaigns or vaccination of individual dogs upon request from owners (on-demand), were conducted quarterly. The implementers pointed out that house-to-house and on-demand vaccination strategies discouraged participation in village-level central point campaigns, as owners reckoned the vaccinators could come to their homes later.

*“Now after the community members knew that after the village-level campaign, there is that of house to house, they are not bringing the dogs again to the central point. They don’t bring the dogs, thinking after the central point the vaccinators will come to their homes” (DLFO IDI, District 1)*

RCs found house-to-house and on-demand vaccination strategies more difficult to implement as they came with higher transport and time demands.

#### Continuous vaccination.

Though house-to-house and on-demand was difficult to implement, the frequent (mostly central point) campaigns provided more vaccination opportunities to dog owners, which was seen to promote high vaccination coverage. The continuous approach through which multiple vaccination pulses happened through the yearly cycle thus gained traction among community members.

*“From my perspective, this program is progressing well. In the past, we used to vaccinate just once and then forget about it. But now, after every three months, they sometimes send messages, asking us to bring our dogs for vaccination” (P9, Community Leaders & Members FGD, District 6).*

#### Thermotolerant vaccine.

The ability to store the Nobivac vaccine in the passive cooling clay pots within communities provided ready access to the vaccine and made the work of the vaccinators easier.

*“Storing the vaccines in the pots has gone well, and when I first arrived, I was surprised at how they can be stored in pots. It turned out that these pots can maintain a cold environment suitable for vaccine storage. The process went well because we followed the necessary procedures” (P1, RCs FGD, District 4).*

#### Resilience of equipment in the field.

Some implementers experienced damage to their passive cooling clay pots and temperature loggers, forcing them to store the vaccines in pots of nearby RCs, disrupting the ready access to vaccines.

*“My experience is similar to the previous speakers. We faced challenges with pots cracking and leaking. To maintain vaccine quality, we needed to keep them in a cool place, which was a challenge. Also, the provided temperature recorders didn’t last long; they would break easily” (P4, RCs FGD, District 2).*

### Domain 5: Governance of CBC-MDV delivery

#### Oversight of implementation.

The DLFOs were supposed to monitor the vaccination campaigns as part of their routine animal health services supervision. Of 45 vaccination teams audited in the third year, 15 said their campaigns were not supervised by a DLFO, which may have contributed to the inactivity of some RCs and OHCs.

Implementers relied on mobile communication in requesting vaccination materials as planned: 38 out of 45 RCs sent the request to the DLFOs via WhatsApp, SMS or phone call; 35 out of 45 RCs still had to travel personally to the district office to collect the materials, while eight RCs received them via a colleague.

#### Policy narratives.

Tanzanian veterinary sector policy stipulates that dogs should be vaccinated at 1,000 TZS shillings per head, whereas vaccination under CBC-MDV was free of charge, which was valued highly by dog owners. However, when asked, implementers and communities postulated the cost of dog vaccination could be successfully shifted to dog owners if preceded by education, introduced progressively, and backed by law, otherwise, the government should provide dog vaccination free of charge.

*“So, if people understand the benefits of the vaccination, they will also accept the costs, and willingly contribute. I believe the community will accept this concept if they receive education before this project ends” (P8, Community Leaders & Members FGD, District 2).*

Community leaders and members suggested that dog vaccination could be effectively enforced by creating a registry of all village dogs.

*“We as livestock keepers will use various methods, including forming groups, to identify those who own dogs and have them join a group” (P5, Community Leaders & Members FGD, District 2).*

However, in several interviews and group discussions, it was thought that dog vaccination campaigns would fail if dog owners were charged.

*“Maybe we should continue seeking sponsors, you know, at the point we’ve reached, people haven’t grasped the importance of vaccinating dogs. So, if we charge no one will bring dogs, to be honest” (P 7, Community Leaders & Members FGD, District 5).*

## Discussion

### Key findings

**Fig 2:**
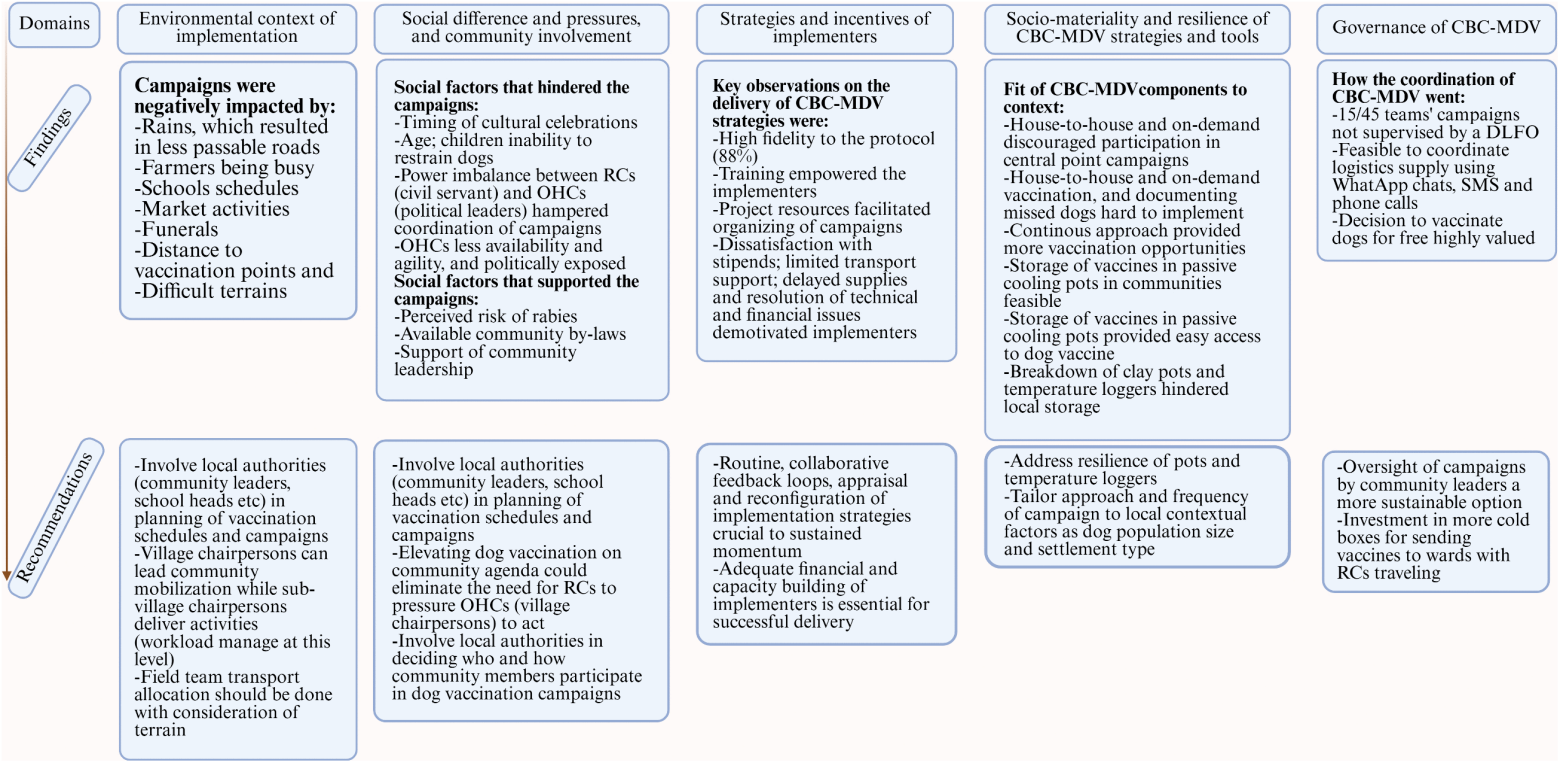
Summary of findings and recommendations for each construct of the socio-anthropological framework.

Interpreting the process evaluation data of CBC-MDV implementation using the socio-anthropological framework showed delivery was impacted by local environmental context, socioeconomic factors, the design of and how implementers delivered the strategies, the suitability of CBC-MDV tools and strategies to the context and how delivery was coordinated. The summary of findings and recommendations for each construct is presented in Fig 2.

### Implications of findings

**Domain 1: Environmental context of implementation:** As reported by other studies [15,23,29], the physical, as well as socioeconomic environments of targeted communities greatly impacted success and should be factored into planning. While rainfall disrupted the campaigns, many people had to bring their dogs after completing farm work or had to wait for children to take the dogs after school, negatively impacting participation in the campaigns (Fig 2). On the other hand, it was widely acknowledged among implementers that school sessions provided effective platforms for disseminating campaign information. This dilemma could be overcome with careful planning of campaigns with school authorities around school sessions, for example, creating awareness through a school-led rabies week celebration and campaigns held the following weekend.

The RCs, community leaders and members all considered the villages to be larger than could be effectively manned by one OHC and advised that each (or group of) sub-village(s) be assigned a vaccination center and vaccination activities be coordinated by Sub-Village Chairpersons. Implementers also advised that terrain and size of villages should be considered when allocating resources (Fig 2). This aligns with findings from the pilot phase of the trial [17].

**Domain 2: Social difference and pressures, and community involvement:** As noted in other studies, cultural activities [16,21] and dog management roles in the household [15,30] should be considered in planning of vaccination campaigns. For example, when vaccination campaign schedules conflicted with cultural activities, people always prioritized the later. Further, dogs tended to trust the person who takes care of them but where this person is very young, restraining the dog for vaccination often became a challenge. Owners’ and vaccinators’ ability to restrain dogs during vaccination is usually a significant challenge in populations that are not used to regular mass dog vaccination campaigns [15,28,31–33]. The organization of vaccination activities itself was impacted by social difference; coordination of the campaigns at ward level was hindered by the power imbalance between OHCs (who wielded more social and political influences) and RCs (simply viewed as government employees). This can be addressed by elevating dog vaccination as a community agenda, where the OHCs perceive it as a desirable goal to pursue for their communities without needing to be persuaded. Alternatively, the assignment of OHCs should consider that they need to be of a rank that RCs can easily instruct. In promoting dog vaccination at community levels, it is important that the right persons are targeted for mobilization and for bringing dogs to vaccination centers.

This study highlighted the importance of considering local sociocultural factors in the planning dog vaccination activities (Fig 2). Indeed, team-led MDV has shown that the diverse socioeconomic contexts of different target communities make it difficult for teams emanating from outside the communities to fully incorporate such factors during planning and, as noted in other mass drug administration campaigns against NTDs amenable to chemotherapy and other community-based interventions, community participation in deciding timing is paramount to minimizing negative impacts of contextual factors [11,34].

Implementers in CBC-MDV observed that involving community leaders helped promote the campaigns and fostered a sense of ownership, which has been reported to be critically important in sustaining NTD programs [35–37]. Communities were consistent about the roles they could play in CBC-MDV, which included advertising campaigns, educating and mobilizing dog owners, and enforcing dog vaccination. These roles are similar to those recorded in other community-based NTD programs [38] and suggest national rabies control programs harness these contributions. However, although communities in Tanzania have social and administrative structures suitable for CBC-MDV delivery, who plays a role and how they are involved needs careful navigation with communities leading the decision processes to prevent persons with vested economic or political interests negatively impacting the community endeavor (11). For example, while the social influence of Village Chairpersons was desirable in mobilizing communities, they were generally seen to be less helpful during implementation due to their advanced age and physical frailty, high social status (compared to the relative lack of kudos associated with working in dog vaccination), poor writing skills, reduced availability (mostly prioritizing other responsibilities over CBC-MDV activities) and inability to strictly enforce dog vaccination because they were politically exposed (Fig 2). Alternatively, RCs opined that using Village Executives (non-elected, government appointees) could help overcome the challenges with Village Chairpersons. Additionally, they suggested that the work required for vaccinating the village dogs would be more manageable if coordinated by Sub-Village Chairpersons. In summary, it is clear that leadership of CBC-MDV at the village level is important and designing how that leadership is structured is crucial to leveraging community factors to promote dog vaccination. Such community factors as the perceived risk of rabies and community leadership that champions knowledge of rabies and enforces existing community laws on dog vaccination (Fig 2) can foster participation.

**Domain 3: Strategies and incentives of implementers:** Factors including intervention design and monetary expectations influenced implementation. It was noted that fidelity to the CBC-MDV protocol improved at the trial phase (compared to the pilot) and that this was attributable to experience and continuous tailoring of the strategies to local context. However, some components, such as the documentation of dogs that missed previous campaigns and the introduction of OHCs to their communities before campaigns, were not delivered as planned (Fig 2) at both the pilot and trial phases [17]. Researchers, implementers and communities need to work together to address why these components were not adopted and how implementation can be improved. The research-implementer-community collaboration is essential during design, implementation and appraisal phases of a community-based intervention. Co-design and collective reconfiguration of strategies ensure co-ownership of the intervention among implementers and targeted communities, with enhanced chances of sustainability.

Additionally, a number of implementers registered frustrations with technical and financial issues, delays in supply of vaccination materials, and having to manage insufficient funds for transport. Similar observations have been made from mass dog vaccination campaigns in other parts of Tanzania and the participation of community drug distributors in onchocerciasis control [15,36] that such issues dampen the morale of field staff and derail implementation efforts. Aside from these frustrations expressed by a number of implementers, the RCs were generally satisfied with organizing vaccination campaigns with the project resources that they had as they reported them to be readily available and sufficient for most of the activities. This suggests that adequate and consistent resourcing of MDV campaigns results in enthusiasm and sends a positive signal on prioritizing rabies control to livestock officers.

**Domain 4: Socio-materiality and resilience of intervention strategies and tools:** The design of CBC-MDV strategies and tools fitted quite well to the context. For example, the thermotolerance of Nobivac Rabies vaccine and the concept of storage in passive cooling clay pots were effective and made dog vaccines easily accessible to ward level livestock officers by facilitating storage of the vaccines in their communities irrespective of availability of electricity. This reduced the requirement for travel to district offices for restocking (Fig 2). Thermotolerance was demonstrably invaluable to the eradication of smallpox [39] and rinderpest [40], and could have similar impact on rabies control. However, there is a need to improve the resilience of the clay pots and temperature loggers to ensure they are reliable in the field.

Additionally, while both implementers and communities thought the continuous vaccination offered more dog vaccination opportunities, implementers advocated scrapping the house-to-house and on-demand strategies, arguing that these strategies were labor-intensive and discouraged participation in central point campaigns. The house-to-house and on-demand strategies were designed to ensure opportunities for dog vaccination was available on an ad hoc basis in between the central point campaigns so that vaccination coverage could be kept high at all times. However, village settlements on the rural landscape of Tanzania are mostly scattered, meaning the vaccinators had to cover great distances to reach a few houses, making these strategies resource-intensive.

**Domain 5: The governance of CBC-MDV delivery:** Overall, the governance of CBC-MDV delivery went well, but as also observed during the pilot phase [17], supervision of the campaigns by DLFOs was limited, possibly contributing to the inactivity of some ward-level vaccinating teams (Fig 2). The supervisory role of DLFOs was limited by availability of resources as they were expected to do this as part of their routine animal health services supervision. Community leaders, if trained and resourced, could provide a less costly and more sustainable supervision of dog vaccination campaigns in their respective villages. This calls for strategic involvement of community leaders in CBC-MDV delivery.

The supply of logistics from district headquarters to wards did not always go as planned. While implementers requested supplies via WhatsApp messaging, they either had to travel personally or depended on colleagues to collect the materials (Fig 2). A type of courier system for transporting consumables from district headquarters to the wards could reduce travel and cost for the RCs, and for the entire district veterinary services if the supply chain could be integrated across all animal disease services. For example, ample cold boxes could be procured to send vaccines and other consumables (and return boxes) via public transport. Given the distances between some of the wards and the district headquarters, such a system could result in considerable time and cost savings for district veterinary services.

#### How CBC-MDV could be optimized.

**Fig 3:**
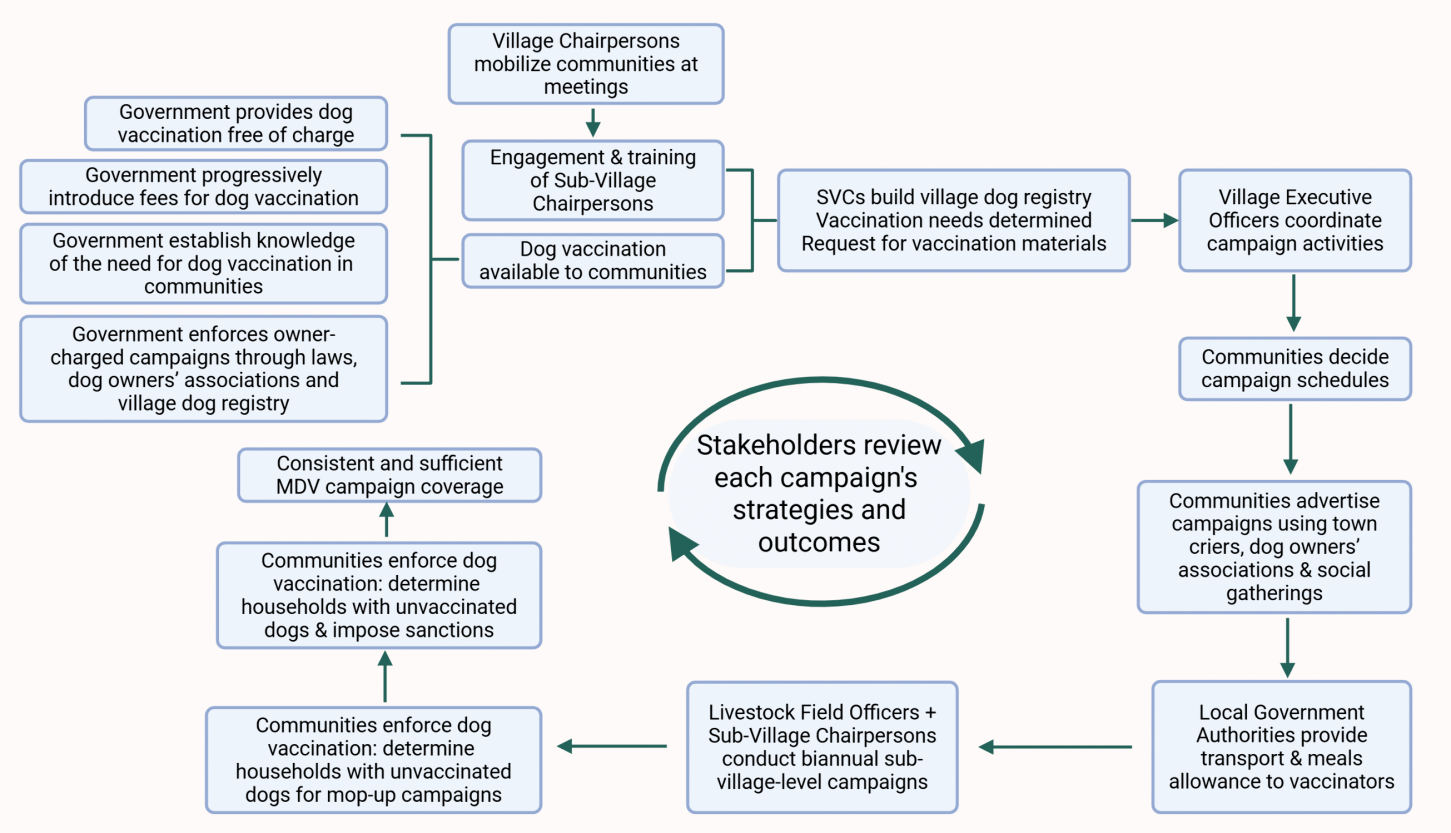
A model of how CBC-MDV could be effectively and sustainably delivered.

We generated a model of how CBC-MDV could be effectively and sustainably delivered beyond the funded research by synthesizing the experiences and reflections (and suggestions) of implementers and communities across all the domains of the socio-anthropological framework (Fig 3). The model highlights how the critical issue of payment for dog vaccination might be integrated into an effective community-led mass dog vaccination campaign strategy, in collaboration with local government and veterinary authorities.

### Usefulness of the socio-anthropological framework in describing implementation success of CBC-MDV

The framework developed by Bardosh [26] was useful for shaping the exploration of key determinants of success and failure of an intervention delivered at the community level. However, Validating the relevance and comprehensiveness of the constructs by experts in community-based interventions could improve the robustness and applicability of the framework. In application, we found some of the domain constructs relatively restrictive as they did not encompass all relevant dimensions or phenomena. For example, in operationalizing Domain 1, we changed the construct name from ‘terrain of intervention’, which implied a focus on just the physical environment, to ‘environmental context of implementation’ as we believed it encompassed more dimensions (Table 1). Using terminologies that are broader in definition for the constructs could also facilitate their interpretation and application across multi-disciplinary fields.

## Conclusions

This study showed that considering environmental, socioeconomic and cultural events of targeted communities is important for successful dog vaccination campaigns. Deeper involvement of communities in planning when and how campaigns are conducted, and in the appraisal of outcomes can significantly address barriers to dog-owner participation, but which community actors and how they are involved need careful consideration. Also, enhancing knowledge of rabies and enforcing by-laws could promote participation. Storing vaccines locally to support continuous dog vaccination at scale was feasible. However, the form of CBC-MDV delivery needed careful tailoring to the local setting. Co-reflexive appraisal of the strategies and support for field implementers through training, resourcing, supervision, responsive resolution of implementation issues, and community involvement would support sustained implementation.

## Supporting information

S1 File Description of CBC-MDV using the TIDieR checklist. (DOCX)

## Abbreviations

CBC-MDV: Community-Based Continuous Mass Dog Vaccination
DLFOs: District Livestock Field Officers (who coordinated campaign logistics at the district level)
OHCs: One Health Champions (were Village Chairpersons and assisted the campaigns at village levels)
LFOs: Livestock Field Officers (animal health officers stationed at the level of a ward – a cluster of 3-5 villages)
LGAs: Local Government Authority
RCT: Randomized Controlled Trial
VEOs: Village Executive Officers (government employees who represent and administer government programs and services at the village level)

## References

[1] Hampson K, Dushoff J, Cleaveland S, Haydon DT, Kaare M, Packer C, et al. Transmission dynamics and prospects for the elimination of canine rabies. PLoS Biol. 2009;7(3):e53. doi: 10.1371/journal.pbio.1000053 19278295 PMC2653555

[2] Mazigo HD, Okumu FO, Kweka EJ, Mnyone LL. Retrospective analysis of suspected rabies cases reported at bugando referral hospital, mwanza, Tanzania. J Glob Infect Dis. 2010;2(3):216–20. doi: 10.4103/0974-777X.68530 20927280 PMC2946675

[3] Cleaveland S, Thumbi SM, Sambo M, Lugelo A, Lushasi K, Hampson K, et al. Proof of concept of mass dog vaccination for thecontrol and elimination of canine rabies. Rev Sci Tech. 2018;37(2):559–68. doi: 10.20506/rst.37.2.2824 30747125 PMC7612386

[4] Lushasi K, Hayes S, Ferguson EA, Changalucha J, Cleaveland S, Govella NJ, et al. Reservoir dynamics of rabies in south-east Tanzania and the roles of cross-species transmission and domestic dog vaccination. J Appl Ecol. 2021;58(11):2673–85. doi: 10.1111/1365-2664.13983 35221371 PMC7612421

[5] Fitzpatrick MC, Hampson K, Cleaveland S, Meyers LA, Townsend JP, Galvani AP. Potential for rabies control through dog vaccination in wildlife-abundant communities of Tanzania. PLoS Negl Trop Dis. 2012;6(8):e1796. doi: 10.1371/journal.pntd.0001796 22928056 PMC3424251

[6] Hampson K, Coudeville L, Lembo T, Sambo M, Kieffer A, Attlan M, et al. Estimating the global burden of endemic canine rabies. PLoS Negl Trop Dis. 2015;9:1–20. doi: 10.1371/journal.pntd.0003709 PMC4400070 25881058

[7] Townsend SE, Sumantra IP, Pudjiatmoko, Bagus GN, Brum E, Cleaveland S, et al. Designing programs for eliminating canine rabies from islands: Bali, Indonesia as a case study. PLoS Negl Trop Dis. 2013;7(8):e2372. doi: 10.1371/journal.pntd.0002372 23991233 PMC3749988

[8] Sambo M, Ferguson EA, Abela-Ridder B, Changalucha J, Cleaveland S, Lushasi K, et al. Scaling-up the delivery of dog vaccination campaigns against rabies in Tanzania. PLoS Negl Trop Dis. 2022;16(2):e0010124. doi: 10.1371/journal.pntd.0010124 35143490 PMC8865671

[9] Lembo T, Hampson K, Kaare MT, Ernest E, Knobel D, Kazwala RR, et al. The feasibility of canine rabies elimination in Africa: dispelling doubts with data. PLoS Negl Trop Dis. 2010;4(2):e626. doi: 10.1371/journal.pntd.0000626 20186330 PMC2826407

[10] Bardosh KL. Towards a science of global health delivery: a socio-anthropological framework to improve the effectiveness of neglected tropical disease interventions. PLoS Negl Trop Dis. 2018;12(7):e0006537. doi: 10.1371/journal.pntd.0006537 30024887 PMC6053127

[11] Mtuy TB, Bardosh K, Ngondi J, Mwingira U, Seeley J, Burton M, et al. Understanding hard-to-reach communities: local perspectives and experiences of trachoma control among the pastoralist Maasai in northern Tanzania. J Biosoc Sci. 2021;53(6):819–38. doi: 10.1017/S0021932020000553 32981544 PMC7618280

[12] Kipanyula MJ. Why has canine rabies remained endemic in the Kilosa district of Tanzania? Lessons learnt and the way forward. Infect Dis Poverty. 2015;4:52. doi: 10.1186/s40249-015-0085-6 26631275 PMC4668701

[13] Knobel DL, Cleaveland S, Coleman PG, Fèvre EM, Meltzer MI, Miranda MEG, et al. Re-evaluating the burden of rabies in Africa and Asia. Bull World Health Organ. 2005;83(5):360–8. 15976877 PMC2626230

[14] Meltzer MI, Rupprecht CE. A review of the economics of the prevention and control of rabies. Part 2: Rabies in dogs, livestock and wildlife. Pharmacoeconomics. 1998;14(5):481–98. doi: 10.2165/00019053-199814050-00003 10344914

[15] Bardosh K, Sambo M, Sikana L, Hampson K, Welburn SC. Eliminating rabies in Tanzania? Local understandings and responses to mass dog vaccination in Kilombero and Ulanga districts. PLoS Negl Trop Dis. 2014;8(6):e2935. doi: 10.1371/journal.pntd.0002935 24945697 PMC4063706

[16] Savadogo M, Soré A-F, Dahourou LD, Ossebi W, Combari AHB, Alambedji RB, et al. Assessing factors associated with owner’s individual decision to vaccinate their dogs against rabies: a house-to-house survey in Ouagadougou, Burkina Faso. Vet World. 2021;14(4):1014–9. doi: 10.14202/vetworld.2021.1014-1019 34083954 PMC8167541

[17] Duamor CT, Hampson K, Lankester F, Lugelo A, Mpolya E, Kreppel K, et al. Development, feasibility and potential effectiveness of community-based continuous mass dog vaccination delivery strategies: lessons for optimization and replication. PLoS Negl Trop Dis. 2022;16(9):e0010318. doi: 10.1371/journal.pntd.0010318 36067231 PMC9481168

[18] Lankester FJ, Wouters PAWM, Czupryna A, Palmer GH, Mzimbiri I, Cleaveland S, et al. Thermotolerance of an inactivated rabies vaccine for dogs. Vaccine. 2016;34(46):5504–11. doi: 10.1016/j.vaccine.2016.10.015 27729174

[19] Lugelo A, Hampson K, Bigambo M, Kazwala R, Lankester F. Controlling human rabies: the development of an effective, inexpensive and locally made passive cooling device for storing thermotolerant animal rabies vaccines. trop Med Infect Dis. 2020;5(3):130. doi: 10.3390/tropicalmed5030130 32796605 PMC7558109

[20] Lugelo A, Hampson K, Ferguson EA, Czupryna A, Bigambo M, Duamor CT, et al. Development of dog vaccination strategies to maintain Herd immunity against Rabies. Viruses. 2022;14(4):830. doi: 10.3390/v14040830 35458560 PMC9028497

[21] Knobel DL, Laurenson MK, Kazwala RR, Boden LA, Cleaveland S. A cross-sectional study of factors associated with dog ownership in Tanzania. BMC Vet Res. 2008;4:5. doi: 10.1186/1746-6148-4-5 18230137 PMC2262882

[22] Sambo M, Lembo T, Cleaveland S, Ferguson HM, Sikana L, Simon C, et al. Knowledge, attitudes and practices (KAP) about rabies prevention and control: a community survey in Tanzania. PLoS Negl Trop Dis. 2014;8(12):e3310. doi: 10.1371/journal.pntd.0003310 25473834 PMC4256472

[23] Sikana L, Lembo T, Hampson K, Lushasi K, Mtenga S, Sambo M, et al. Dog ownership practices and responsibilities for children’s health in terms of rabies control and prevention in rural communities in Tanzania. PLoS Negl Trop Dis. 2021;15(3):e0009220. doi: 10.1371/journal.pntd.0009220 33690720 PMC7946275

[24] Hoffmann TC, Glasziou PP, Boutron I, Milne R, Perera R, Moher D, et al. Better reporting of interventions: template for intervention description and replication (TIDieR) checklist and guide. BMJ. 2014;348:g1687. doi: 10.1136/bmj.g1687 24609605

[25] Changalucha J, Anderson D, Yoder J, Stone B, Lugelo A, Kimera S, et al. Evaluating delivery models for mass dog vaccination: a cost-effectiveness analysis of community-led and team-led vaccination strategies for rabies control in resource-limited settings. Front Trop Dis. 2026;6. doi: 10.3389/fitd.2025.1725443

[26] Bardosh KL. Towards a science of global health delivery: a socio-anthropological framework to improve the effectiveness of neglected tropical disease interventions. PLoS Negl Trop Dis. 2018;12(7):e0006537. doi: 10.1371/journal.pntd.0006537 30024887 PMC6053127

[27] Mtuy TB, Bardosh K, Ngondi J, Mwingira U, Seeley J, Burton M, et al. Understanding hard-to-reach communities: local perspectives and experiences of trachoma control among the pastoralist Maasai in northern Tanzania. J Biosoc Sci. 2021;53(6):819–38. doi: 10.1017/S0021932020000553 32981544 PMC7618280

[28] Duamor CT, Hampson K, Lankester F, Lugelo A, Mpolya E, Kreppel K, et al. Development, feasibility and potential effectiveness of community-based continuous mass dog vaccination delivery strategies: Lessons for optimization and replication. PLoS Negl Trop Dis. 2022;16(9):e0010318. doi: 10.1371/journal.pntd.0010318 36067231 PMC9481168

[29] Castillo-Neyra R, Brown J, Borrini K, Arevalo C, Levy MZ, Buttenheim A, et al. Barriers to dog rabies vaccination during an urban rabies outbreak: qualitative findings from Arequipa, Peru. PLoS Negl Trop Dis. 2017;11(3):e0005460. doi: 10.1371/journal.pntd.0005460 28306717 PMC5371379

[30] Duamor CT, Lankester F, Mpolya E, Ferguson EA, Johnson PC, Wyke S, et al. Participation in mass dog vaccination campaigns in Tanzania: benefits of community engagement. Front Public Health. 2022;10:971967. doi: 10.3389/fpubh.2022.971967 36311637 PMC9616113

[31] Walwa BW, Kimaro EG, Mpolya EA, Sambo M. Factors associated with poor compliance to rabies post-exposure prophylaxis among dog bite victims in Maswa District in Tanzania: a cross-sectional study. PAMJ-OH. 2024;14. doi: 10.11604/pamj-oh.2024.14.2.41309

[32] Sambo M, Hampson K, Johnson PCD, Johnson OO. Understanding and overcoming geographical barriers for scaling up dog vaccinations against rabies. Sci Rep. 2024;14(1):30975. doi: 10.1038/s41598-024-82085-4 39730865 PMC11681037

[33] Duamor CT, Lankester F, Mpolya E, Ferguson EA, Johnson PC, Wyke S, et al. Participation in mass dog vaccination campaigns in Tanzania: Benefits of community engagement. Front Public Health. 2022;10:971967. doi: 10.3389/fpubh.2022.971967 36311637 PMC9616113

[34] CDI Study Group. Community-directed interventions for priority health problems in Africa: results of a multicountry study. Bull World Health Organ. 2010;88(7):509–18. doi: 10.2471/BLT.09.069203 20616970 PMC2897985

[35] Cleaveland S, Lankester F, Townsend S, Lembo T, Hampson K. Rabies control and elimination: a test case for One Health. Vet Rec. 2014;175(8):188–93. doi: 10.1136/vr.g4996 25172649 PMC7612423

[36] Amazigo UV, Leak SGA, Zoure HGM, Okoronkwo C, Diop Ly M, Isiyaku S, et al. Community-directed distributors-The “foot soldiers” in the fight to control and eliminate neglected tropical diseases. PLoS Negl Trop Dis. 2021;15(3):e0009088. doi: 10.1371/journal.pntd.0009088 33661903 PMC7932156

[37] Madon S, Malecela MN, Mashoto K, Donohue R, Mubyazi G, Michael E. The role of community participation for sustainable integrated neglected tropical diseases and water, sanitation and hygiene intervention programs: a pilot project in Tanzania. Soc Sci Med. 2018;202:28–37. doi: 10.1016/j.socscimed.2018.02.016 29501716 PMC5906643

[38] Duamor CT, Datchoua-Poutcheu FR, Chounna Ndongmo WP, Yoah AT, Njukang E, Kah E, et al. Programmatic factors associated with the limited impact of Community-Directed Treatment with Ivermectin to control Onchocerciasis in three drainage basins of South West Cameroon. PLoS Negl Trop Dis. 2017;11(11):e0005966. doi: 10.1371/journal.pntd.0005966 29155826 PMC5714394

[39] Henderson DA, Klepac P. Lessons from the eradication of smallpox: an interview with D. A. Henderson. Philos Trans R Soc Lond B Biol Sci. 2013;368(1623):20130113. doi: 10.1098/rstb.2013.0113 23798700 PMC3720050

[40] Mariner JC, House JA, Mebus CA, Sollod AE, Chibeu D, Jones BA, et al. Rinderpest eradication: appropriate technology and social innovations. Science. 2012;337(6100):1309–12. doi: 10.1126/science.1223805 22984063

