# Supplementary material for: Implementing mass dog rabies vaccination through a community-based continuous approach: A socio-anthropological process evaluation
Source: PLoS Negl Trop Dis. 2026 Jun 30;20(6):e0014091. doi: 10.1371/journal.pntd.0014091 (PMC13372228; doi:10.1371/journal.pntd.0014091)
Supplement: S1 File — (DOCX) [file pntd.0014091.s001.docx]

Description of CBC-MDV using the TIDieR checklist

| **Item** | **Intervention description** |
| --- | --- |
| Brief name of intervention | Community-based continuous mass dog vaccination (CBC-MDV) approach |
| Why the intervention | CBC-MDV was designed to provide continuous access to mass dog vaccination to rural communities in Tanzania by storing vaccines at ward levels and using them to conduct quarterly campaigns and make vaccination available upon request from dog owners. The aim is to ensure that herd immunity is maintained among the dog population all year round by   1. consistently vaccinating dogs across all communities; 2. reaching dogs missed out during main campaigns and newly bought/ born dogs during inter-campaign intervals |
| What materials were used in the delivery of the intervention | Nobivac^TM^ Canine Rabies vaccine and vaccination consumables (syringes, needles, gloves, alcohol, cotton wool etc) and cold boxes for carrying vaccines and trunks for storing these vaccination materials  Smartphones with data-capturing applications installed, vaccination cards/ certificates, vaccination registers and an implementation manual to guide the delivery  Branded caps and T-shirts, megaphones to support advertising of campaigns, and gumboots and raincoats for use on rainy days  Locally made clay passive cooling pots for storing the vaccines in the wards, and temperature loggers to monitor the internal temperature of the pots |
| Procedures, activities or processes used | The key intervention procedures included:  District Veterinary Officers (DVOs) or District Livestock Field Officers (DLFOs) at district levels were assigned to coordinate the vaccination activities to empower them and get their buy-in for this new approach  (*DLFOs are experienced animal health officers who have at least a diploma and are assigned the role of coordinating district veterinary services in the absence of a veterinarian*)  At the ward level, LFOs were assigned the role of rabies vaccination coordinator and, under the authority of the DLFO / DVO, coordinated all rabies vaccination activities in the villages of their ward. The vaccinator was known as the Rabies Coordinator (RC).  A single village level One Health Champion (OHC) was chosen from the community in each village. The OHCs tended to be the Village Chairperson. The OHC was responsible for assisting the RC during the vaccination activities in their village. This included estimating dog population numbers in the village, assisting with advertising, and assisting with vaccinating dogs and cats during the campaign.  Community leaders were to be involved in the planning and delivery of rabies vaccination activities at village levels to maximize community support for the programme and to ensure activities were coordinated around community activities by incorporating community knowledge of the local context  The RCs and OHCs were to attend village meetings to explain the new approach to mass dog vaccination and how it would be carried out, and roles the community and individual dog owners were expected to play in the process. These engagement activities were to start 7-10 days before clinics to ensure the communities become aware of the need for dog vaccination and the campaign schedules  Vaccines were stored in clay passive cooling pots at the RCs house, which was typically located in one of the villages of the ward (wards typically comprise 3-4 villages).  Vaccination campaigns were offered free of charge to dog owners to encourage participation  To try to ensure participatory monitoring, the research team received daily (on days when dogs were vaccinated), weekly and monthly reports on vaccination activities through data being uploaded by the RCs from their data capturing applications. After each round of (quarterly) campaigns, the research team provided feedback to the RCs on the outcome of vaccination activities, the research team, vaccinators and communities discussed the campaign activities and made recommendations.  Campaign procedures:  Vaccines were available for use throughout the year. Vaccination pulses were organized in each village every three months starting with a pulse hosted at the central point of each village at the beginning of the first quarter; over the following two months, the OHCs were responsible for compiling a list of the dogs that had missed the village-level campaigns and those that were born or acquired afterward. The owners of these dogs were encouraged to attend vaccination pulses hosted at month 3, 6 and 9. The vaccination pulses in months 3, 6 and 9 were typically carried out at a central point of each sub-village within each village (a village typically has 3 – 5 sub-villages). In addition, dog owners could request that their dogs be vaccinated (on demand) at other times of the year (in between the pulses).  A single pulse of vaccination activities within a quarter typically involved the following activities:   - DVO / DLFO receives vaccine & vaccination materials - OHCs estimate dog populations in their villages for the first round of campaigns. For subsequent campaigns, they determine the number of dogs missed in previous campaign(s). - RCs request vaccine and vaccination materials to allow vaccination of 80% of the dog population of their ward from the DVO / DLFOs office - The RCs coordinate the delivery of materials from the DVO / DLFO’s office to their wards - RCs and OHCs advertise the campaigns through use of a megaphone and posters - RCs and OHCs conduct vaccination campaigns at central locations of each village, or sub-village or at the household level - RCs upload vaccination records after each day’s vaccination activities via the data collection app and provide weekly and monthly reports on other vaccination activities within the quarter to the research team by taking photographs of pages of their vaccination logbooks - The OHCs compile list of dogs that missed the vaccination by visiting houses or after receiving a call from dog owners who have dogs that need vaccination - RCs and OHCs target these dogs either through sub-village level clinics or house-to-house visitation at the end of months 3, 6 and 9 - After every six months, unused vaccines were to be returned to the DVO / DLFO office for safe disposal - The research team, vaccinators and communities review delivery after each round of campaigns: the research team provides feedback to communities through the RCs and OHCs and receives responses from communities through the same channel. |
| Who provided the intervention | DVOs / DLFOs who received managed stocks of vaccines and vaccination materials, and monitored the campaigns in their districts  RCs who coordinated campaigns in their wards, and were responsible for vaccinating dogs and capturing their biodata  OHCs (mainly village chairpersons) were responsible for engaging their communities on rabies and advertising campaigns  (While the *DVOs/ DLFOs, LFOs performed their roles on CBC-MDV as part of their routine duties, the OHCs were selected based on their influence as village chairpersons. All the implementers received allowances to support their work)* |
| What was the mode of delivery of the intervention | Community engagement and sensitization were conducted face-to-face as part of village meetings  Advertising of vaccination campaigns was carried out through megaphone announcements in the villages, at village gatherings and by pasting notices at vantage points such as the village government offices  The dog vaccinations were delivered face-to-face  Monitoring of vaccination activities by DVOs / DLFOs and the research team was done through electronically uploaded data  Reviews of vaccination activities by vaccinators and their communities were carried out through village meetings |
| Where the intervention was delivered | CBC-MDV was delivered in randomly selected wards (n=56) in all six administrative districts of the Mara region of Tanzania. These districts consist of rural, agropastoral communities in the lake zone, in the north-west of the country. |
| When and how often was the intervention was delivered | One round of vaccination campaigns was organized every three months, using village-level central point clinics in the first quarter and either sub-village level clinics or house-to-house visitation during subsequent quarters. In between these, the RCs also visited houses to vaccinate dogs anytime upon a request made by owners |
| Aspects of the intervention that were subject to tailoring | The RCs were able to plan vaccination schedules and the actual method of delivery to suit the schedule of their other work commitments, and to suit the number of dogs that required vaccination and the size of the villages in their wards. For example, RCs could elect to vaccinate through house-to-house if the number of dogs that required vaccinating suggested that this would be a more efficient method. |
| How the intervention was modified in delivery | The protocol was modified in the following ways:   - Due to inactivity between the vaccination pulses, the DVOs / DLFOs (supported by the research team) instructed the RCs that they were expected to also locate and vaccinate unvaccinated dogs during the period between the pulses. To encourage this, approximate targets were set for each village based on the estimate of how many dogs remained unvaccinated. - To be continued as data analysis progresses… |
| Planned: How intervention adherence or fidelity was assessed and by whom | The implementation process was guided by a manual  Fidelity was measured as part of a process evaluation of the implementation by an implementation scientist designated for that purpose |
| Actual: extent to which intervention was delivered as planned | To be assessed when data analysis in completed … |
