# Supplementary material for: Implementing mass dog rabies vaccination through a community-based continuous approach: A socio-anthropological process evaluation
Source: PLoS Negl Trop Dis. 2026 Jun 30;20(6):e0014091. doi: 10.1371/journal.pntd.0014091 (PMC13372228; doi:10.1371/journal.pntd.0014091)
Supplement: Attachment — Submitted filename: Response to Reviewers.docx [file pntd.0014091.s003.docx]

F33-LHD-v19.1

*Please, reply to:* Christian Tetteh Duamor

**Location:** DAR ES SALAAM

**Ref. 003** IHI/HSIEP/2026

May 28, 2026

PLOS NTDs

Dear Editor,

|  | RESPONSE TO YOURS AND REVIEWERS’ COMMENTS |
| --- | --- |

Following yours and the reviewers’ comments and suggestions, we have revised the manuscript as outlined below: the reviewers’ comments are presented then followed by our responses.

**Reviewer #1**

**Comment 1**: Rabies is a dangerous disease. Most patients who develop symptomatic rabies die, so prevention is extremely important. Therefore, I believe this study is very valuable. However, the authors could further clarify the following points.

The authors are requested to clearly specify the study design. If this is a randomized controlled trial (RCT), how was it conducted, and what was its impact on the outcomes? If it is not an RCT, but rather a descriptive study on the role of the community and local authorities in rabies prevention conducted alongside an RCT, this should be clearly stated. In that case, the authors should also describe how the RCT was implemented and clarify the roles of the community and local authorities in each study group.

The variables to be collected in the study and their specific definitions.

**Response:** Two words have been added to clarity the study type employed in the process evaluation of the CBC-MDV arm of the RCT; it is clearly stated in the introduction and description of the intervention in the methods that this study is focused on CBC-MDV arm and described only this in detail as the Team-led arm is extensively described in the literature as referenced.

**Comment 2**: In the Results section, what do the contents of domains 1, 2, 3, 4, and 5 represent, and how do they contribute to the study? They appear to function like a summary table of factors influencing the effectiveness of the vaccination program. However, are there differences in the percentages across regions? If so, do these differences affect the uptake of rabies vaccination in those areas? It seems more like a record of opinions collected from the survey rather than analytical results.

**Response**: We would like to highlight that the results from the exploration of implementation using the socio-anthropological framework is purely qualitative; additionally, the objective of this study is focused on evaluating the implementation processes and what influenced that as opposed to impact or outcome evaluation as done in a different paper submitted for publication by Lankester et al., [25].

**Comment 3:** In the Discussion section, the authors summarize the findings; however, I do not see sufficient numerical data or supporting explanations to substantiate the points they make. And in the absence of numerical data, it becomes scientifically less convincing to readers.

**And**

**Comment 4**: I believe that improving the effectiveness of rabies prevention is extremely important; therefore, this is a meaningful and necessary topic. However, if this study focuses on the impact of a socio-anthropological process evaluation, the authors need to clearly demonstrate how such an evaluation has influenced rabies prevention (i.e., vaccination uptake), with specific variables and quantitative data. This would help to better illustrate the importance of the socio-anthropological approach.

**Response**: We wish to highlight once again that the socio-anthropological framework domains mainly examine determinants of implementation success through qualitative exploration, as such, our study is largely qualitative process evaluation as opposed to impact or outcome and should be assessed in that light. Within the context of rabies elimination, the last mile for endemic communities in least developed countries is mainly the understanding of how, what should be considered and who should be involved in planning, delivery and governance of mass dog vaccination campaigns to sustain successful implementation. This has been the focus of evaluating the implementation processes of this new way of conducting mass dog vaccination campaigns (community-based continuous mass dog vaccination approach), to see how it should be operationalized to ensure it is, first of all delivered well.

**Reviewer #2**

**Comment 1**: This study employs a mixed-methods design and demonstrates a strong qualitative analytical component. The authors achieve reasonable clarity in presenting the overall framework and analysis plan. However, there appears to be some conceptual overlap between Domains 2 and 3, particularly regarding aspects of community involvement.

In addition, the coding process is not described with sufficient clarity. It remains unclear how the coding framework was developed and how it was systematically aligned with the socio-anthropological framework guiding the analysis.

**Response**: We have now edited how we adapted the socio-anthropological framework; we would like to highlight that domain 2 focuses on influence of social difference and how communities were involved while domain 3 focuses on fidelity to the protocol by the implementers and what influenced the implementation choices. It also might be helpful to mention that this arm was community-based and not community-led or implemented.

The description of how the data was coded is also revised to make how the code frame was developed and systematically aligned to socio-anthropological framework.

**Comment 2**: The proposed model (Figure 3) is difficult to follow, as it includes multiple actors and processes, making the overall message unclear. In particular, it is not evident how the model contributes to the optimization of vaccination strategies.

I understood the main message of the article as highlighting that “social science is not finding its way into practice.” In this sense, failures in implementation appear to be driven by micro-level structures in the field, with gaps at this level reflected across the five domains presented in the framework.

**Response**: The proposed model (Figure 3) is now revised to show how that model represents a more potent approach to mass dog vaccination.

**Reviewer #3**

**Comment 1**: While the conclusions are supported by the presented data, the limitations of the analysis are not sufficiently articulated. In particular, the applied framework does not account for additional factors that may influence the success of the vaccination program, including dog owners’ perceived behavioral control, attitudes, and challenges in handling dogs during vaccination campaigns. These limitations should be explicitly discussed.

It would be helpful to review the references once more for accuracy. For example, in lines 749–750, the journal name is missing. Similar issues appear in lines 707–708 and 771–773

Sarah Cleaveland is acknowledged as having contributed to the writing, review, and editing of the manuscript; however, her name is not listed among the co-authors. Could you please clarify the reason for her exclusion from the authorship list?

**Response**: Dog owners perceived behavioral of rabies control, attitudes, and challenges in handling dogs during vaccination campaigns are now discussed under domain 2 in the discussion.

We have also worked on the references the reviewer identified to have errors except reference number 25 which is still under review and do not have the journal in the bibliography, and have reviewed the author contributions.

**Comment 2**: This study focused on assessing what impacted the implementation processes of the CBC-MDV approach using the socio-anthropological framework. It is a valuable approach to design strategy in improving vaccination coverage in the future. This study showed that considering environmental, socioeconomic and cultural events of targeted communities is important for successful dog vaccination campaigns. Deeper involvement of communities in planning when and how campaigns are conducted, and in the appraisal of outcomes can significantly address barriers to dog-owner participation. Therefore, the results are an important contribution to the knowledge of decision making to design the most efficient and effective strategies for improving dog vaccination coverage in endemic rabies areas like Tanzania. However, the presentation and the accuracy of the paper should be improved and the references need to be double checked before it published.

**Response**: On the issues of accuracy of the findings presented, we confirm that these are accurate representation of the findings based on the data we collected, we have read the manuscript again, matching the results with our conclusions and believed the results do support the conclusions

We appreciate your consideration and look forward to hearing from you.

With best regards,

Christian Tetteh DUAMOR, PhD

(For other co-authors)
