# Supplementary material for: Implementing mass dog rabies vaccination through a community-based continuous approach: A socio-anthropological process evaluation
Source: PLoS Negl Trop Dis. 2026 Jun 30;20(6):e0014091. doi: 10.1371/journal.pntd.0014091 (PMC13372228; doi:10.1371/journal.pntd.0014091)
Supplement: Peer review history

# Peer review: Decision Letter 0

**DOI:** 10.1371/journal.pntd.0014091.r001

**Authors:** Richard Bowen, Annapaola Rizzoli

22 Apr 2026

PNTD-D-26-00339

Implementing mass dog rabies vaccination through a continuous community-based approach: a socio-anthropological process evaluation

PLOS Neglected Tropical Diseases

Dear Dr. Duamore,

Thank you for submitting your manuscript to PLOS Neglected Tropical Diseases. After careful consideration, we feel that it has merit but does not fully meet PLOS Neglected Tropical Diseases’s publication criteria as it currently stands. Therefore, we invite you to submit a revised version of the manuscript that addresses the points raised during the review process.

Please submit your revised manuscript by 21th May 2026. If you will need more time than this to complete your revisions, please reply to this message or contact the journal office at plosntds@plos.org. When you're ready to submit your revision, log on to https://www.editorialmanager.com/pntd/ and select the 'Submissions Needing Revision' folder to locate your manuscript file.

Please include the following items when submitting your revised manuscript:

* A letter that responds to each point raised by the editor and reviewer(s). You should upload this letter as a separate file labeled 'Response to Reviewers'. This file does not need to include responses to any formatting updates and technical items listed in the 'Journal Requirements' section below.

* A marked-up copy of your manuscript that highlights changes made to the original version. You should upload this as a separate file labeled 'Revised Manuscript with Track Changes'.

* An unmarked version of your revised paper without tracked changes. You should upload this as a separate file labeled 'Manuscript'.

If you would like to make changes to your financial disclosure, competing interests statement, or data availability statement, please make these updates within the submission form at the time of resubmission. Guidelines for resubmitting your figure files are available below the reviewer comments at the end of this letter.

We look forward to receiving your revised manuscript.

Kind regards,

Richard A. Bowen, DVM PhD

Academic Editor

PLOS Neglected Tropical Diseases

Annapaola Rizzoli

Section Editor

PLOS Neglected Tropical Diseases

Shaden Kamhawi

co-Editor-in-Chief

PLOS Neglected Tropical Diseases

orcid.org/0000-0003-4304-636XX

Paul Brindley

co-Editor-in-Chief

PLOS Neglected Tropical Diseases

orcid.org/0000-0003-1765-0002

**Additional Editor Comments:**

Your manuscript has been reviewed by 3 experts in the rabies field and each considered it a valuable contribution. One reviewer provided some minor suggestions for clarifying a few items - please evaluate those comments, edit your manuscript as you think best and resubmit along with a short "responses to reviewers" document. Thank you.

**Journal Requirements:**

If the reviewer comments include a recommendation to cite specific previously published works, please review and evaluate these publications to determine whether they are relevant and should be cited. There is no requirement to cite these works unless the editor has indicated otherwise.

1) Please provide an Author Summary. This should appear in your manuscript between the Abstract (if applicable) and the Introduction, and should be 150-200 words long. The aim should be to make your findings accessible to a wide audience that includes both scientists and non-scientists. Sample summaries can be found on our website under Submission Guidelines:

https://journals.plos.org/plosntds/s/submission-guidelines#loc-parts-of-a-submission

2) Please upload all main figures as separate Figure files in .tif or .eps format. For more information about how to convert and format your figure files please see our guidelines:

https://journals.plos.org/plosntds/s/figures

3) We have noticed that you have uploaded Supporting Information files, but you have not included a list of legends. Please add a full list of legends for your Supporting Information files after the references list.

4) In the online submission form, you indicated that All data on which discussion and conclusions are based are available for upon request.. All PLOS journals now require all data underlying the findings described in their manuscript to be freely available to other researchers, either

1. In a public repository

2. Within the manuscript itself

3. Uploaded as supplementary information.

This policy applies to all data except where public deposition would breach compliance with the protocol approved by your research ethics board. If your data cannot be made publicly available for ethical or legal reasons (e.g., public availability would compromise patient privacy), please explain your reasons by return email and your exemption request will be escalated to the editor for approval. Your exemption request will be handled independently and will not hold up the peer review process, but will need to be resolved should your manuscript be accepted for publication. One of the Editorial team will then be in touch if there are any issues.

5) Please amend your detailed Financial Disclosure statement. This is published with the article. It must therefore be completed in full sentences and contain the exact wording you wish to be published.

1) State what role the funders took in the study. If the funders had no role in your study, please state: "The funders had no role in study design, data collection and analysis, decision to publish, or preparation of the manuscript."

2) If any authors received a salary from any of your funders, please state which authors and which funders..

If you did not receive any funding for this study, please simply state: u201cThe authors received no specific funding for this work.u201d

6) Some material included in your submission may be copyrighted. According to PLOS’s copyright policy, authors who use figures or other material (e.g., graphics, clipart, maps) from another author or copyright holder must demonstrate or obtain permission to publish this material under the Creative Commons Attribution 4.0 International (CC BY 4.0) License used by PLOS journals. Please closely review the details of PLOS’s copyright requirements here: PLOS Licenses and Copyright. If you need to request permissions from a copyright holder, you may use PLOS's Copyright Content Permission form.

Please respond directly to this email and provide any known details concerning your material's license terms and permissions required for reuse, even if you have not yet obtained copyright permissions or are unsure of your material's copyright compatibility. Once you have responded and addressed all other outstanding technical requirements, you may resubmit your manuscript within Editorial Manager.

Potential Copyright Issues:

i) Figure 1: please (a) provide a direct link to the base layer of the map (i.e., the country or region border shape) and ensure this is also included in the figure legend; and (b) provide a link to the terms of use / license information for the base layer image or shapefile. We cannot publish proprietary or copyrighted maps (e.g. Google Maps, Mapquest) and the terms of use for your map base layer must be compatible with our CC BY 4.0 license.

Note: if you created the map in a software program like R or ArcGIS, please locate and indicate the source of the basemap shapefile onto which data has been plotted.

If your map was obtained from a copyrighted source please amend the figure so that the base map used is from an openly available source. Alternatively, please provide explicit written permission from the copyright holder granting you the right to publish the material under our CC BY 4.0 license.

If you are unsure whether you can use a map or not, please do reach out and we will be able to help you. The following websites are good examples of where you can source open access or public domain maps:

* U.S. Geological Survey (USGS) - All maps are in the public domain. (http://www.usgs.gov)

* PlaniGlobe - All maps are published under a Creative Commons license so please cite “PlaniGlobe, http://www.planiglobe.com, CC BY 2.0” in the image credit after the caption. (http://www.planiglobe.com/?lang=enl)

* Natural Earth - All maps are public domain. (http://www.naturalearthdata.com/about/terms-of-use/)

**Reviewers' comments:**

Reviewer's Responses to Questions

**Key Review Criteria Required for Acceptance?**

As you describe the new analyses required for acceptance, please consider the following:

**Methods**

-Are the objectives of the study clearly articulated with a clear testable hypothesis stated?

-Is the study design appropriate to address the stated objectives?

-Is the population clearly described and appropriate for the hypothesis being tested?

-Is the sample size sufficient to ensure adequate power to address the hypothesis being tested?

-Were correct statistical analysis used to support conclusions?

-Are there concerns about ethical or regulatory requirements being met?

Reviewer #1: Rabies is a dangerous disease. Most patients who develop symptomatic rabies die, so prevention is extremely important. Therefore, I believe this study is very valuable. However, the authors could further clarify the following points.

The authors are requested to clearly specify the study design. If this is a randomized controlled trial (RCT), how was it conducted, and what was its impact on the outcomes? If it is not an RCT, but rather a descriptive study on the role of the community and local authorities in rabies prevention conducted alongside an RCT, this should be clearly stated. In that case, the authors should also describe how the RCT was implemented and clarify the roles of the community and local authorities in each study group.

The variables to be collected in the study and their specific definitions.

Reviewer #2: This study employs a mixed-methods design and demonstrates a strong qualitative analytical component. The authors achieve reasonable clarity in presenting the overall framework and analysis plan. However, there appears to be some conceptual overlap between Domains 2 and 3, particularly regarding aspects of community involvement.

In addition, the coding process is not described with sufficient clarity. It remains unclear how the coding framework was developed and how it was systematically aligned with the socio-anthropological framework guiding the analysis.

Reviewer #3: -Are the objectives of the study clearly articulated with a clear testable hypothesis stated? YES

-Is the study design appropriate to address the stated objectives? YES

-Is the population clearly described and appropriate for the hypothesis being tested? YES

-Is the sample size sufficient to ensure adequate power to address the hypothesis being tested? YES

-Were correct statistical analysis used to support conclusions? NA

-Are there concerns about ethical or regulatory requirements being met? No

**********

**Results**

-Does the analysis presented match the analysis plan?

-Are the results clearly and completely presented?

-Are the figures (Tables, Images) of sufficient quality for clarity?

Reviewer #1: In the Results section, what do the contents of domains 1, 2, 3, 4, and 5 represent, and how do they contribute to the study? They appear to function like a summary table of factors influencing the effectiveness of the vaccination program. However, are there differences in the percentages across regions? If so, do these differences affect the uptake of rabies vaccination in those areas? It seems more like a record of opinions collected from the survey rather than analytical results.

Reviewer #2: The analysis presented matched the analysis plan

The results are clearly presented with good examples of each domain. The topics structure is clear in all domains.

Reviewer #3: The analysis presented match the analysis plan and the results are clearly and completely presented.

**********

**Conclusions**

-Are the conclusions supported by the data presented?

-Are the limitations of analysis clearly described?

-Do the authors discuss how these data can be helpful to advance our understanding of the topic under study?

-Is public health relevance addressed?

Reviewer #1: In the Discussion section, the authors summarize the findings; however, I do not see sufficient numerical data or supporting explanations to substantiate the points they make. And in the absence of numerical data, it becomes scientifically less convincing to readers.

Reviewer #2: The proposed model (Figure 3) is difficult to follow, as it includes multiple actors and processes, making the overall message unclear. In particular, it is not evident how the model contributes to the optimization of vaccination strategies.

I understood the main message of the article as highlighting that “social science is not finding its way into practice.” In this sense, failures in implementation appear to be driven by micro-level structures in the field, with gaps at this level reflected across the five domains presented in the framework.

Reviewer #3: While the conclusions are supported by the presented data, the limitations of the analysis are not sufficiently articulated. In particular, the applied framework does not account for additional factors that may influence the success of the vaccination program, including dog owners’ perceived behavioral control, attitudes, and challenges in handling dogs during vaccination campaigns. These limitations should be explicitly discussed.

It would be helpful to review the references once more for accuracy. For example, in lines 749–750, the journal name is missing. Similar issues appear in lines 707–708 and 771–773

Sarah Cleaveland is acknowledged as having contributed to the writing, review, and editing of the manuscript; however, her name is not listed among the co-authors. Could you please clarify the reason for her exclusion from the authorship list?

**********

**Editorial and Data Presentation Modifications?**

Use this section for editorial suggestions as well as relatively minor modifications of existing data that would enhance clarity. If the only modifications needed are minor and/or editorial, you may wish to recommend “Minor Revision” or “Accept”.

Reviewer #1: (No Response)

Reviewer #2: (No Response)

Reviewer #3: (No Response)

**********

**Summary and General Comments**

Use this section to provide overall comments, discuss strengths/weaknesses of the study, novelty, significance, general execution and scholarship. You may also include additional comments for the author, including concerns about dual publication, research ethics, or publication ethics. If requesting major revision, please articulate the new experiments that are needed.

Reviewer #1: I believe that improving the effectiveness of rabies prevention is extremely important; therefore, this is a meaningful and necessary topic. However, if this study focuses on the impact of a socio-anthropological process evaluation, the authors need to clearly demonstrate how such an evaluation has influenced rabies prevention (i.e., vaccination uptake), with specific variables and quantitative data. This would help to better illustrate the importance of the socio-anthropological approach.

Reviewer #2: (No Response)

Reviewer #3: This study focused on assessing what impacted the implementation processes of the CBC-MDV approach using the socio-anthropological framework. It is a valuable approach to design strategy in improving vaccination coverage in the future. This study showed that considering environmental, socioeconomic and cultural events of targeted communities is important for successful dog vaccination campaigns. Deeper involvement of communities in planning when and how campaigns are conducted, and in the appraisal of outcomes can significantly address barriers to dog-owner participation. Therefore, the results are an important contribution to the knowledge of decision making to design the most efficient and effective strategies for improving dog vaccination coverage in endemic rabies areas like Tanzania. However, the presentation and the accuracy of the paper should be improved and the references need to be double checked before it published.

**********

PLOS authors have the option to publish the peer review history of their article (what does this mean?). If published, this will include your full peer review and any attached files.

If you choose “no”, your identity will remain anonymous but your review may still be made public.

**Do you want your identity to be public for this peer review?**  For information about this choice, including consent withdrawal, please see our Privacy Policy.

Reviewer #1: No

Reviewer #2: **Yes:** FABIO GHILARDI

Reviewer #3: No

**Figure resubmission:**

While revising your submission, we strongly recommend that you use PLOS’s NAAS tool (https://ngplosjournals.pagemajik.ai/artanalysis) to test your figure files. NAAS can convert your figure files to the TIFF file type and meet basic requirements (such as print size, resolution), or provide you with a report on issues that do not meet our requirements and that NAAS cannot fix.

After uploading your figures to PLOS’s NAAS tool - https://ngplosjournals.pagemajik.ai/artanalysis, NAAS will process the files provided and display the results in the "Uploaded Files" section of the page as the processing is complete. If the uploaded figures meet our requirements (or NAAS is able to fix the files to meet our requirements), the figure will be marked as "fixed" above. If NAAS is unable to fix the files, a red "failed" label will appear above. When NAAS has confirmed that the figure files meet our requirements, please download the file via the download option, and include these NAAS processed figure files when submitting your revised manuscript.

**Reproducibility:**

To enhance the reproducibility of your results, we recommend that authors of applicable studies deposit laboratory protocols in protocols.io, where a protocol can be assigned its own identifier (DOI) such that it can be cited independently in the future. Additionally, PLOS ONE offers an option to publish peer-reviewed clinical study protocols. Read more information on sharing protocols at https://plos.org/protocols?utm_medium=editorial-email&utm_source=authorletters&utm_campaign=protocols

# Author comment: Author response to Decision Letter 1

**DOI:** 10.1371/journal.pntd.0014091.r002

30 May 2026

Attachment Submitted filename: Response to Reviewers.docx

# Editor report: Decision Letter 1

**DOI:** 10.1371/journal.pntd.0014091.r003

**Authors:** Richard Bowen, Annapaola Rizzoli, Richard Bowen, Max Eyre

2 Jun 2026

Dear Duamor,

We are pleased to inform you that your manuscript 'Implementing mass dog rabies vaccination through a community-based continuous approach: a socio-anthropological process evaluation' has been provisionally accepted for publication in PLOS Neglected Tropical Diseases.

Before your manuscript can be formally accepted you will need to complete some formatting changes, which you will receive in a follow up email. A member of our team will be in touch with a set of requests.

Please note that your manuscript will not be scheduled for publication until you have made the required changes, so a swift response is appreciated.

IMPORTANT: The editorial review process is now complete. PLOS will only permit corrections to spelling, formatting or significant scientific errors from this point onwards. Requests for major changes, or any which affect the scientific understanding of your work, will cause delays to the publication date of your manuscript.

Should you, your institution's press office or the journal office choose to press release your paper, you will automatically be opted out of early publication. We ask that you notify us now if you or your institution is planning to press release the article. All press must be co-ordinated with PLOS.

Thank you again for supporting Open Access publishing; we are looking forward to publishing your work in PLOS Neglected Tropical Diseases.

Best regards,

Max Thomas Eyre, PhD

Section Editor

PLOS Neglected Tropical Diseases

Max Eyre

Section Editor

PLOS Neglected Tropical Diseases

Shaden Kamhawi

co-Editor-in-Chief

PLOS Neglected Tropical Diseases

orcid.org/0000-0003-4304-636XX

Paul Brindley

co-Editor-in-Chief

PLOS Neglected Tropical Diseases

orcid.org/0000-0003-1765-0002

***********************************************************

Thank you for the thoughtful revisions to your manuscript in response to reviewer comments.

p.p1 {margin: 0.0px 0.0px 0.0px 0.0px; line-height: 16.0px; font: 14.0px Arial; color: #323333; -webkit-text-stroke: #323333}span.s1 {font-kerning: none

# Editor report: Acceptance letter

**DOI:** 10.1371/journal.pntd.0014091.r004

**Authors:** Richard Bowen, Annapaola Rizzoli, Richard Bowen, Max Eyre

Dear Duamor,

We are delighted to inform you that your manuscript, "

Implementing mass dog rabies vaccination through a community-based continuous approach: a socio-anthropological process evaluation," has been formally accepted for publication in PLOS Neglected Tropical Diseases.

We have now passed your article onto the PLOS Production Department who will complete the rest of the publication process. All authors will receive a confirmation email upon publication.

The corresponding author will soon be receiving a typeset proof for review, to ensure errors have not been introduced during production. Please review the PDF proof of your manuscript carefully, as this is the last chance to correct any scientific or type-setting errors. Please note that major changes, or those which affect the scientific understanding of the work, will likely cause delays to the publication date of your manuscript. Note: Proofs for Front Matter articles (Editorial, Viewpoint, Symposium, Review, etc...) are generated on a different schedule and may not be made available as quickly.

Soon after your final files are uploaded, the early version of your manuscript will be published online unless you opted out of this process. The date of the early version will be your article's publication date. The final article will be published to the same URL, and all versions of the paper will be accessible to readers.

For Research Articles, you will receive an invoice from PLOS for your publication fee after your manuscript has reached the completed accept phase. If you receive an email requesting payment before acceptance or for any other service, this may be a phishing scheme. Learn how to identify phishing emails and protect your accounts at https://explore.plos.org/phishing.

Thank you again for supporting open-access publishing; we are looking forward to publishing your work in PLOS Neglected Tropical Diseases.

Best regards,

Shaden Kamhawi

co-Editor-in-Chief

PLOS Neglected Tropical Diseases

Paul Brindley

co-Editor-in-Chief

PLOS Neglected Tropical Diseases
